# Lipid Peroxidation and Antioxidative Capacity Are Unaltered in Transitional Breast Milk Exposed to Light from Women Giving Birth to Preterm Infants before 32 Weeks of Gestation

**DOI:** 10.3390/nu15122818

**Published:** 2023-06-20

**Authors:** Moa Norrgrann, Malin Hörnfeldt, Faiza Latheef, Ylva Thernström Blomqvist, Anders Larsson, Mattias Paulsson, Barbro Diderholm

**Affiliations:** 1 Department of Women’s and Children’s Health, Uppsala University, University Children’s Hospital, 751 85 Uppsala, Sweden; moa.norrgrann@akademiska.se (M.N.); f.latheef87@gmail.com (F.L.); ylva.thernstrom_blomqvist@kbh.uu.se (Y.T.B.); mattias.paulsson@kbh.uu.se (M.P.); 2 Department of Medical Sciences, Clinical Chemistry, Uppsala University, Uppsala University Hospital, 751 85 Uppsala, Sweden; anders.larsson@akademiska.se

**Keywords:** human breast milk, preterm infants, naso-gastric tube feeding, enteral nutrition, phototherapy, lipid peroxidation, malondialdehyde, 4-hydroxynonenal, total antioxidant capacity, light protection

## Abstract

Breast milk (BM) is the primary nutrition for infants and has a high content of lipids. Preterm infants receive expressed BM via tube feeding, and they are frequently treated with phototherapy. When parenteral nutrition (PN) is exposed to light and/or phototherapy, lipid peroxidation (LPO) increases. By light-protecting PN, morbidity and mortality are reduced in preterm infants through the reduction of oxidative stress. We aimed to investigate whether light-protecting breast milk could reduce LPO. Twelve mothers giving birth to a preterm infants of less than 32 weeks of gestational age were included. Transitional BM was collected and divided into three study groups; light-protected, ward light and phototherapy light. Baseline samples were collected after expression and the exposures started within one hour. Feeding syringe samples were exposed to light for 30 up to 360 min. Nasogastric tube samples were run through a tube under the same light conditions. Samples were stored in −80 °C until analyses of malondialdehyde (MDA), 4-hydroxynonenal (4-HNE) and total antioxidant capacity (TAC). There were no significant differences in MDA, 4-HNE or TAC levels observed between the different study groups. This study indicates that the light exposure of expressed transitional BM does not affect LPO and the levels of MDA, 4-HNE or TAC.

## 1. Introduction

Breast milk is recommended as the primary nutrition for all infants, including preterm and sick infants, during their first 6 months of life [1]. It has many benefits compared to donated breast milk and formula, including antioxidant capacity [2,3,4,5]. The composition of breast milk changes throughout the different stages of lactation with colostrum (1–3 days postpartum), transitional (4–14 days) and mature breast milk (>14 days) and there is a difference in content between milk from women giving birth to term or preterm infants [6]. The major source of energy in breast milk comes from fat, with a content of more than 200 different fatty acids both saturated and unsaturated [7].

Lipid peroxidation (LPO) is the oxidative degradation of lipids, predominantly affecting polyunsaturated fatty acids due to their chemical structure, with end products such as malondialdehyde (MDA) and 4-hydroxynonenal (4-HNE) [8]. Both MDA and 4-HNE are reliable and frequently used markers for lipid peroxidation in previous studies [9]. The constant level of LPO in the human body generates a substantial amount of reactive aldehydes or free radical species; however, detoxification through various types of antioxidants can keep these concentrations at a low level [10]. Both enzymatic and non-enzymatic antioxidants as well as hormones with antioxidant capacities have been found to be present in breast milk [11].

Preterm infants are prone to oxidative stress due to low antioxidant capacity, with both low enzyme activity and low levels of vitamin E and C [8,12]. In addition to this, neonates are exposed to situations in the perinatal period, such as hypoxia and hyperoxia [13], mechanical ventilation [14] and phototherapy [15], that can lead to an increased production of free radicals. It is currently known that oxidative stress may have a significant role to play in the pathogenesis of various neonatal conditions, such as infant respiratory distress syndrome (IRDS), necrotizing enterocolitis, bronchopulmonary dysplasia, late-onset sepsis and retinopathy of prematurity. At present, there are no clinical guidelines recommending the use of antioxidants to prevent or treat these conditions [12,13,16].

In a recent study from 2020, preterm infants with IRDS had higher levels of the LPO product MDA as well as lower total antioxidant capacity (TAC) in serum compared to controls [17]. Analogous results have been achieved in previous studies on RDS and oxidative stress markers [18].

Studies have shown that parenteral nutrition (PN) with a lipid content that has been exposed to light, especially phototherapy, has an increased presence of LPO products. The formation of peroxides can be limited by shielding PN bags and administration sets from light [19,20]. A meta-analysis by Chessex et al. [21], including more than 800 preterm infants, concluded that light-protecting PN led to a 50% reduction in mortality. This publication has led to the development of new guidelines recommending the light protection of PN bags and administration devices to children under the age of two, and for intravenous lipid emulsions administered to preterm infants [22,23].

Despite the recommendation of light-protecting PN, there are only a few studies measuring the effect of light and phototherapy exposure on expressed breast milk and the possible increased risk of LPO [24,25]. At present, there are no clinical guidelines regarding light-protecting breast milk when administered via tube feeding. The current guideline at the Uppsala University Children’s Hospital allows for breast milk to be stored at room temperature in bottles or feeding syringes for up to 6 h.

The aim of this study was to examine whether light exposure alters the concentrations of LPO markers and/or the antioxidant capacity in transitional breast milk with the hypothesis of an increase in lipid peroxidation markers and a decrease in antioxidative capacity. 

## 2. Materials and Methods

This observational cross-sectional study was conducted at the Neonatal Intensive Care Unit (NICU), University Children’s Hospital, Uppsala and the Department of Women’s and Children’s Health, Uppsala University, Sweden. 

### 2.1. Subjects 

**Figure 1:**
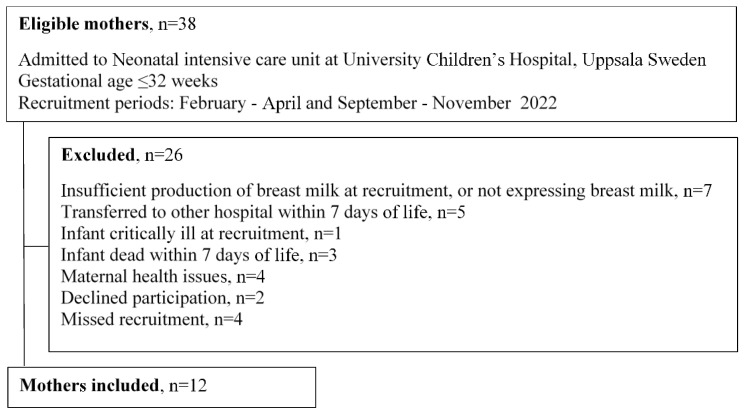
Recruitment diagram.

Twelve consecutive mothers were recruited to the study between February and November 2022, with a halt in data collection between June to September due to a lack of staff during the summer (Figure 1). 

The inclusion criteria were women who had given birth to preterm infants that were under 32 weeks of gestational age and were admitted to the NICU for more than one week. On the day of sampling, the mother had to have sufficient breast milk production that a donation of breast milk would not impact the amount/quantity required by the infant. Exclusion criteria were insufficient breast milk production within 14 days post-partum, the preterm infant being critically ill, mothers being younger than 18 years old and mothers with an infection such as hepatitis, HIV or colonized with multi-resistant bacteria.

### 2.2. Collection of Breast Milk

The participating mothers expressed 30–50 mL of breast milk with an electric breast pump (Symphony double electric breast pump, Medela AG, Baar, Switzerland) at day 6–13 postpartum between 07.00–10.00 in the morning. The breast pump was set to a fixed program with a duration of 15 min, pumping from both breasts simultaneously. The breast milk was expressed into a plastic breast milk bottle (polypropylene/thermoplastic elastomers, Medela AG, Baar, Switzerland) of 80–150 mL, connected to the breast pump. The bottle was light-protected with aluminum foil. The milk was then kept at room temperature for between 15–45 min before being exposed to light in various degrees.

### 2.3. Light Exposure of Breast Milk

#### 2.3.1. Light Exposure Measurements

Light settings between and during days were measured using a light meter (Velleman DEM300: mini digital light meter, Gavere, Belgium). Meteorological data were accessed from SMHI, the Swedish Meteorological and Hydrological Institute (www.smhi.se, accessed on 3 April 2023) for the study period. The parameter “Radiation—solar hours” denoted the number of seconds of sunlight per hour was used. The data were downloaded, sorted to match the time frame and further calculations were performed using Microsoft Excel (version 2016). The station used was Stockholm (98735).

#### 2.3.2. Syringe Samples Kept at Room Temperature

The breast milk was aliquoted into sixteen 1 mL syringes (VY-P syringe for Nutrisafe2 enteral connection, Vygon, Ecouen, France) and capped during the experiment (Nutrisafe2 syringe cap, Vygon, Ecouen, France). 

First a light-protected control sample of 1 mL was immediately divided into two specimens of 0.5 mL and added to tubes (Micro tube 2 mL, Polypropylene PP, with screw cap, Sarstedt AG & Co., Nümbrecht, Germany). 

Fifteen samples of 1 mL each were categorized according to light exposure:

A. 5 × 1 mL syringes light-protected with aluminum foil.
B. 5 × 1 mL syringes exposed to standard daytime lighting in the NICU.
C. 5 × 1 mL syringes exposed to phototherapy with two light sources: one was 38 cm above and one was directly underneath (Giraffe^®^ Blue Spot PT Lite, GE Healthcare, Chicago, IL, USA and BiliSoft^®^ Blue LED Fiberoptic Blanket Phototherapy, GE Healthcare, Chicago, IL, USA, respectively).

The 1 mL syringes, filled with breast milk, were either exposed or protected from light for 30 min, 1, 2, 3 or 6 h. After being exposed to or protected from light, the 1 mL breast milk from each syringe was divided into two specimens of 0.5 mL and added to a 2 mL test tube each. Before dividing the breast milk, the syringes were manually homogenized. 

#### 2.3.3. Nasogastric Tube Administration Simulation

The nasogastric feeding tube (VYGON, Nutrisafe2, Polyurethane, PUR, 06 Fr, Internal diameter: 1.5 mm, Length 75 cm) with an extension set (VYGON, Nutrisafe2, Polyvinylchloride PVC, 06 Fr, Internal diameter: 1.5 mm, Length 150 cm) was connected to a 10 mL feeding syringe. The 10 mL syringe was then connected to a syringe pump (BD Alaris^®^ Enteral Syringe Pump, Becton, Dickinson and Company, Franklin Lakes, NJ, USA) with a set infusion rate of 9 mL/h, mimicking the clinical routine when administering continuous feeding in a preterm infant.

First a control sample was immediately run through the light-protected tube system and divided into two test tubes of 2 mL. Then, the pump was started at the set speed and two samples of 2 mL each were either light-protected or exposed to standard daytime lighting in the NICU or to phototherapy, with a total of six samples. 

All samples described in Section 2.3.2 and Section 2.3.3 were frozen immediately after each light-exposure setting and stored at −70 °C, awaiting further analysis.

### 2.4. Breast Milk Quantification of Lipid Peroxides, Antioxidants and Macro-Nutrient Content

The analysis of breast milk samples were performed at the Clinical Chemistry and Pharmacology Akademiska Laboratory, Uppsala University Hospital, after data collection was completed. A total of 480 milk samples (40 per included study subject) were analyzed for malondialdehyde (MDA), 4-hydroxynonenal (4-HNE) and total antioxidant capacity (TAC). Considering that every milk sample was a duplicate, the mean value of the two was calculated. All assays were performed blinded without clinical information about the samples.

#### 2.4.1. Lipid Peroxides

##### Malondialdehyde

All samples were analyzed using a lipid peroxidation (MDA) assay kit (Colorimetric/Fluorometric) (ab118970), Abcam, UK. Analysis via colorimetric assay was performed according to the instructions of the manufacturer [26]. The absorbance was measured using a SpectraMax 250 (Molecular Devices, Sunnyvale, CA, USA). The concentrations in the samples were determined by comparing the optical density of the sample with the standard curve. 

##### 4-Hydroxynonenal

The samples were analyzed using a human 4-HNE (4-hydroxynonenal) competitive-ELISA Kit (EKX-W727S9-96, Nordic Biosite, Täby, Sweden) [27]. The assay was performed according to the instructions of the manufacturer. Bound 4-HNE was detected via a biotinylated detection antibody and an HRP-streptavidine. The absorbance was measured using a SpectraMax 250 reader (Molecular Devices) and the concentration in the samples was determined by comparing the absorbance of the samples to the standard curve. 

#### 2.4.2. Total Antioxidant Capacity

The analysis was performed using a commercial colorimetric plate reader analysis (Colorimetric Total Antioxidant Capacity Assay Kit (ab65329), Abcam, Cambridge, UK). Analysis was performed according to the instructions provided by the manufacturer [28]. Samples were diluted 50–100 times to allow for analysis in the standard curve range. 

#### 2.4.3. Macro-Nutrient Content

As per clinical routine, macro-nutrient content (protein, fat and carbohydrates) was analyzed in all breast milk from the participating mothers using a Miris Human Milk Analyser™ (Miris Holding, Uppsala, Sweden) via mid-infrared transmission spectroscopy [29,30]. 

### 2.5. Statistical Analysis

Statistical analysis was performed using SPSS (Version 28, SPSS^®^ IBM^®^ Statistics, Armonk, NY, USA). Maternal and infant characteristics as well as macro-nutrient contents of the BM were described as mean, standard deviation, median and range. The levels of MDA, 4-HNE and TAC were described by mean values and standard deviation. A paired *t*-test was used to compare the baseline with samples with different light exposures. To determine the association between macro-nutrient content and LPO markers, Pearson’s correlation test was performed. A one-way analysis of variance (ANOVA) was used to analyze whether there were any associations between all four study conditions, including the baseline sample and differences between all time and light settings. Statistical significance was considered at *p* < 0.05.

## 3. Results

### 3.1. Subjects

Twelve mothers were enrolled in the study (Figure 1). Maternal and infant characteristics are displayed in Table 1. Seven mothers were multipara and none of their previous children were born preterm. The different conditions preceding the preterm births were as follows: four cases of the premature rupture of membranes (PPROM), including one case of chorioamnionitis; four cases of preeclampsia where one case developed into HELPP (hemolysis, elevated liver enzymes and low platelet count) syndrome; one case of intrauterine growth restriction with affected placental function; one case of uterine rupture; and one case of twin to twin transfusion syndrome in conjunction with the stillbirth of one twin.

### 3.2. Breast Milk Analysis

#### 3.2.1. Lipid Peroxides

##### MDA Syringe Samples

**Figure 2:**
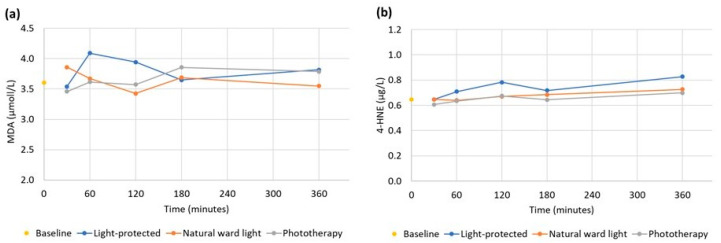
(**a**) Mean malondialdehyde (MDA) (µmol/L) and (**b**) 4-hydroxynonenal (4-HNE) (µg/L) in syringe samples during different durations and types of light exposures.

The mean MDA concentration in the baseline syringe sample was 3.5 ± 1.1 µmol/L. The MDA levels at the different durations of light exposures are displayed in Figure 2a. There were no significant changes in MDA levels in the syringe samples at different durations of exposures to the standard daytime lighting of the NICU or phototherapy light compared to light-protected samples tested via one-way ANOVA (30 min: *p* = 0.71, 1 h: *p* = 0.59, 2 h: *p* = 0.63, 3 h: *p* = 0.99, 6 h: *p* = 0.85). Paired *t*-tests of MDA concentrations comparing the baseline level to each light condition at 6 h showed no statistically significant differences (light-protected: *p* = 0.21; ward light: *p* = 0.78; phototherapy: *p* = 0.51). 

##### MDA Nasogastric Tube Samples

The mean concentration of MDA at baseline was 3.4 ± 1.2 µmol/L; light-protected 3.5 ± 1.3 µmol/L; room/daylight 3.5 ± 1.3 µmol/L and phototherapy 3.9 ± 1.3 µmol/L. Although concentrations of MDA were somewhat higher in the phototherapy group, one-way ANOVA showed no differences between the groups (*p* = 0.78).

##### 4-HNE Syringe Samples

The average concentration of 4-HNE in the baseline samples was 0.65 ± 0.27 µg/L. The 4-HNE levels at the different durations of light exposure are presented in Figure 2b. There were no significant changes in the 4-HNE levels in the syringe samples when comparing the different durations of exposures with room/daylight or phototherapy light with light-protected samples using one-way ANOVA (30 min: *p* = 0.92, 1 h: *p* = 0.78, 2 h: *p* = 0.70, 3 h: *p* = 0.86, 6 h: *p* = 0.79). Comparing 4-HNE at baseline with concentrations at each light condition after 6 h of exposures, using paired *t*-tests, showed no statistically significant differences (light-protected: *p* = 0.61; ward light: *p* = 0.46; phototherapy: *p* = 0.26). 

##### 4-HNE Nasogastric Tube Samples

The average concentration of 4-HNE at baseline was 0.71 ± 0.27 µg/L; light-protected 0.71 ± 0.38 µg/L; room/daylight 0.68 ± 0.33 µg/L; and phototherapy 0.62 ± 0.27 µg/L. Although concentrations of 4-HNE were somewhat higher in the phototherapy group, one-way ANOVA showed no differences between the groups (*p* = 0.90).

#### 3.2.2. Total Antioxidative Capacity

##### TAC Syringe Samples

**Figure 3:**
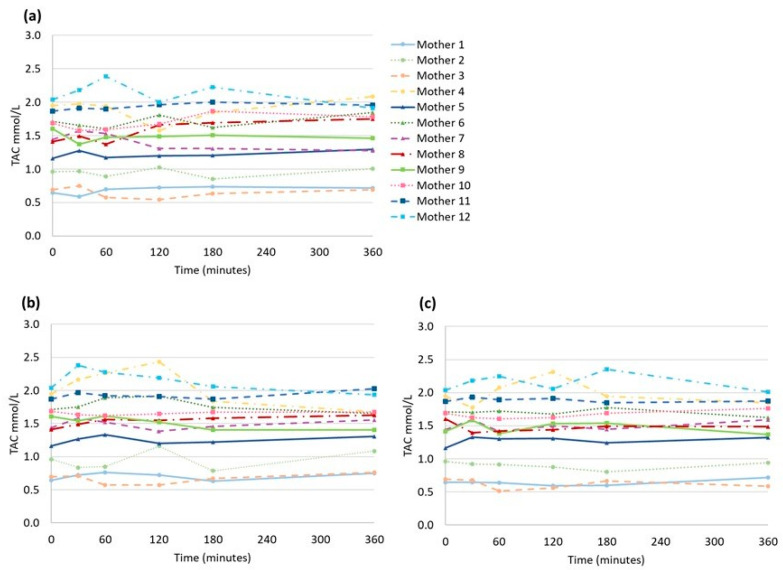
Total antioxidant capacity (TAC) (mmol/L) of each breast milk sample in syringes from the 12 mothers under different types of light exposures (**a**) light-protected, (**b**) ward light and (**c**) phototherapy.

The mean baseline level of TAC was 1.43 ± 0.47 mmol/L. There were no statistically significant differences comparing TAC during the three different light conditions at each time point (30 min: *p* = 0.94, 1 h: *p* = 0.90, 2 h: *p* = 0.89, 3 h: *p* = 0.97, 6 h: *p* = 0.96) using a one-way ANOVA. Paired *t*-tests of the TAC comparing the baseline to each light condition at 6 h showed no statistically significant differences (ward light: *p* = 0.61; phototherapy: *p* = 0.91). There was a large variation in TAC between mothers in the study. However, for each mother, there was little variation in the TAC over time at the varying levels of light exposure (Figure 3). 

##### TAC Nasogastric Tube Samples

The average concentration of TAC at baseline was 1.50 ± 0.46 mmol/L; light-protected 01.50 ± 0.50 mmol/L; room/daylight 1.47 ± 0.52 mmol/L and phototherapy 1.43 ± 0.49 mmol/L. While TAC was higher at baseline in the control group, one-way ANOVA did not show any statistically significant difference between any of the three light conditions or between any of the three light conditions and the baseline samples (*p* = 0.98). The biggest difference was observed between the baseline and phototherapy sample, but this was not statistically significant when calculated with a paired *t*-test (*p* = 0.08).

#### 3.2.3. Macro-Nutrient Content in the Breast Milk

There was moderate correlation between the lipid content of the individual breast milk and the MDA, 4-HNE and TAC baseline samples. For lactose only minimal, or no, linear correlations were seen (Table 2).

### 3.3. Light Meter Results

**Figure 4:**
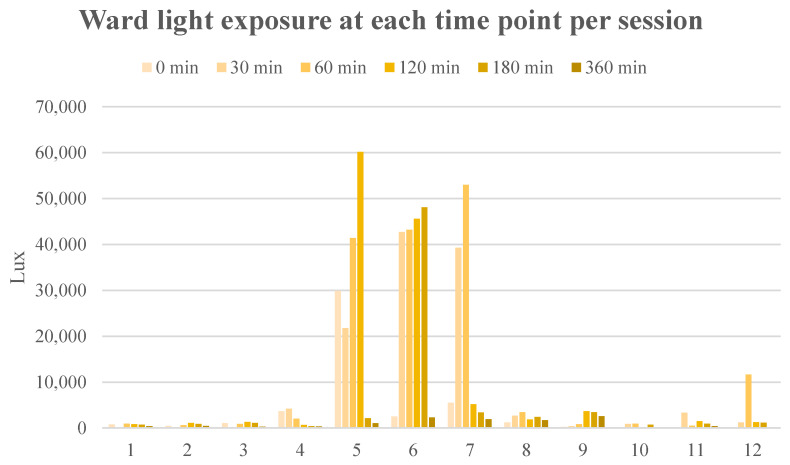
Ward light exposure for each time point for each participating mother.

Ward light varied greatly between sessions and within each session (min 42 Lux and max 60200 Lux), as illustrated in Figure 4. The Lux values for mothers 5, 6, and 7 were especially high compared to the other mothers. These sessions were conducted during the spring and on sunny days. During the year 2022, the number of sun hours per day based on meteorological observations varied from 0 to 18 h/d, depending on time of year and cloudiness. Studies on mothers 1 and 8 were carried out on cloudy days with 0 h sun radiation.

## 4. Discussion

In this study, we examined the effects of different types of light exposure on the presence of lipid peroxides and total antioxidative capacity in transitional breast milk from women giving birth to preterm infants born before 32 weeks of gestation. Breast milk in syringes used for tube feeding were either exposed to standard daytime lighting in the NICU or phototherapy light or were light-protected during different time periods between 30 min up to 6 h. In addition, breast milk was pumped slowly through a gavage tube with the same light conditions to mimic the clinical situation when feeding an extremely preterm infant.

The analysis of two different lipid peroxides MDA and 4-HNE showed stable levels during both room/daylight and phototherapy light conditions up to 6 h as well as during transport through a gavage tube as compared to light-protected samples. Similarly, there was no increased breakdown of small antioxidant molecules included in the analysis of TAC under the same light conditions. These results indicate the stability of human breast milk handled in the clinical setting relating to the potential risk of reactive aldehyde formation and total antioxidant capacity. These results negate the hypothesis in this study that light exposure over time would increase the levels of lipid peroxides and decrease total antioxidative capacity in preterm transitional breast milk.

### 4.1. Lipid Peroxides

The baseline level of MDA was somewhat higher in our study and our results on 4-HNE at baseline was comparable to that of Nessel et al. [31] who compared donor human milk with term and preterm milk by measuring lipid peroxidation markers including MDA and 4-HNE. The infants included in their study were somewhat more mature (30 weeks + 6 days vs. 28 weeks + 2 days) and there was no information on the type of breast milk in relation to the time after delivery. Several plausible factors could explain the high baseline of MDA in our samples, for example, levels of endogenously produced peroxides in the mother.

The levels of MDA and 4-HNE in our study were unaltered by exposure to room temperature and different types of light up to a duration of 6 h. Others have studied the levels of the markers of oxidative stress after exposure to room/daylight and/or phototherapy light [24,25]. A study by van Zoeren-Grobben et al. [25] compared breast milk to formula and the effects of both storage time and phototherapy on lipid peroxidation by measuring free linoleic acid and its hydroperoxide. They found no increase in lipid peroxidation markers in breast milk that had been exposed to phototherapy [25]. However, Unal et al. [24] showed an unaltered total oxidant status measured by mmol H_2_O_2_ per liter in preterm breast milk but a reduced total antioxidative capacity in breast milk (discussed below), resulting in an increased oxidative stress index in milk exposed to phototherapy light for 20 min. 

We have not found any previous study that tested the levels of 4-HNE in breast milk after different light exposures. Our data show that storage at room temperature and exposure to standard ward lighting/daylight or phototherapy light for up to 6 h will not affect the production of 4-HNE.

Comparing our results with previous research on parenteral nutrition [32], it can be seen that the levels of MDA are in the same range; however, different analytical methods and lipid contents compared to breast milk have been used. The levels of 4-HNE in our study are considerably lower than the levels found by others in earlier research on parenteral nutrition [33].

### 4.2. Total Antioxidant Capacity

There are some data on the levels of TAC in breast milk after preterm delivery at various GA and at different postnatal ages. Most data on preterm milk are from mothers giving birth to moderately preterm infants born at 32 weeks of gestation or later. In our study, the infants were born between 24 to 31 weeks of gestation, with a median of 28 weeks. The levels of TAC in breast milk in our study at day 9 was somewhat lower (TAC 1.43 mmol/L at baseline) in comparison with data on preterm milk from Păduraru et al. [34] TAC 2.03 mmol /L) at day 7 (median GA = 32), and Akdag et al. [35] (TAC 3.2 mmol/L) at day 4 (mean GA = 31). 

Our data on TAC in preterm transitional breast milk show that there is a great inter-individual difference in levels between the lactating women, but that the intra-individual levels of TAC were stable between baseline and up to 6 h of exposure to room temperature and light protection as well as room/daylight or phototherapy light. 

In contrast, Unal et al. found that breast milk exposed to phototherapy had significantly lower TAC levels compared to control samples. One difference between the studies is that the control samples in Unal’s study were not light-protected but exposed to natural light [24]. Another difference between the studies is that the breast milk in their study was stored in 5 mL syringes, compared to 1 mL syringes in our study. The surface, in relation to volume and the potential area of exposure to phototherapy light, was proportionally larger in our study. Furthermore, we explored the effect of phototherapy light on TAC in breast milk administered through a nasogastric tube, with an even greater surface to volume relation, without any change in TAC.

### 4.3. Macro-Nutrient Content

Fat is the most highly variable macro-nutrient of milk [6] and varies both diurnally as well as during a single session of expression. A standardized expressing technique according to clinical routine was used to achieve the same conditions for each participating mother, and the breast milk expressed in the morning was collected from each mother to reduce the diurnal effects on its composition. The inter-individual differences in MDA, 4-HNE and TAC could only partially be explained by the different lipid contents of the samples, and no correlation was seen with the hydrophilic contents, such as lactose, in breastmilk. In contrast to Nessel et al. [31], we did not perform a thorough analysis of the fatty acid spectrum in breast milk. Our assay only provided an approximate level of lipid content and did not consider a more detailed analysis of the relation to specific fatty acids and their different properties.

### 4.4. Storage Time/Light Conditions

The breast milk in this study was stored at −80 °C while awaiting analysis in order to best preserve its antioxidant capacity [35]. However, one study showed that levels of MDA increase even at −80 °C if breast milk is stored for more than 30 days [36]. With regard to TAC, there are conflicting results as to whether storage time changes the concentrations over time [37]. There was no correlation between storage time and the level of TAC in our study. A study including 44 participants showed that antioxidative capacity was reduced for full-term transitional milk and mature milk after storage at −80 °C for two months, while no difference existed for colostrum [37]. Another study of 98 participants showed no significant difference for preterm colostrum stored at −80 °C for three months [35]. Ideally, for future research, the analysis of TAC should be conducted on the breast milk immediately after light exposure in order to avoid potentially altering the MDA, TAC and, potentially, 4-HNE values due to storage conditions. 

In clinical practice, breast milk is not exposed to direct phototherapy for 6 h, but possibly to up to six hours at room temperature, as well as standard ward lighting/daylight. In this study, we aimed to examine the potential maximum impact of different light settings and how this could possibly affect the concentrations of lipid peroxides and TAC over time. We mimicked existing clinical practice in this study by measuring MDA, 4-HNE and TAC concentrations in the feeding syringe and the nasogastric tube samples, as breast milk is exposed to either standard ward lighting in the NICU or phototherapy during administration. 

### 4.5. Strengths and Weaknesses

Twelve mothers were included in this study, which can be seen as a relatively low number of participants, thereby limiting the statistical power of the study. The aim was to examine the potential maximum impact of light exposure in a setting as close to the clinical routine as possible, which meant acquiring 30–50 mL of breast milk from each mother. As mentioned in the exclusion criteria for this study, several potential participants could not be enrolled due to limits in the production of breast milk. If a future study design could require a smaller amount of milk, it could be possible to include a larger study population. Another weakness was sample handling with the breast milk not being directly analyzed after the expression but instead frozen. This could have affected the baseline level of the markers.

A strength of this study was the analysis of transitional breast milk exclusively from mothers who delivered preterm infants. This group of infants was chosen since they are the most vulnerable and at increased risk of being exposed to oxidative stress. As shown in studies on parenteral nutrition and light exposure [21], preterm infants can benefit the most from a decrease in oxidative load via light-protection.

## 5. Conclusions

In conclusion, we found high levels of MDA and 4-HNE in all study protocol samples, and no significant decrease when light-protected. Several plausible factors could explain the high baseline of MDA, for example endogenous produced peroxides in the mother. In contrast to one previous study, we did not find any change in TAC concentrations, regardless of light exposure or time. 

However, further studies are essential to examine whether light-protecting breast milk in a similar manner to PN is necessary to reduce lipid peroxidation and thereby reduce oxidative stress in preterm infants. This relates particularly to the time between milk expression and analysis, which could be shortened to avoid unnecessary oxygen and light exposure. Further studies could use a study design such as ours and include more patients to increase statistical power. Another recommendation would be to conduct a randomized control trial measuring mortality/morbidity, similarly to the studies examining light exposure and parenteral nutrition [21]. 

## Figures and Tables

**Table 1:** Maternal and infant characteristics.

	Mean (SD)	Median (Range)	n (%)
Maternal characteristics:			
Age (years)	29.7 (6.6)	28.5 (18–42)	-
Pre-pregnancy BMI	26.2 (8.4)	22.7 (17.3–42)	-
Mode of delivery cesarean section			10 (83%)
Infant characteristics:			
Girls	-	-	8 (66%)
Weight (g)	1095 (435)	1038 (545–1717)	-
Length (cm)	36.3 (3.9)	36.5 (30–41)	-
Head circumference (cm)	25.8 (3.3)	26 (20–31)	-
Gestational age (weeks + days)	28 + 1 (2 + 2)	28 + 2 (24 + 5–31 + 0)	-
Postnatal age at study (days)	9.1 (2.2)	9 (6–13)	-

**Table 2:** Macro-nutrient (lipid, protein, carbohydrates/lactose and energy) content in the breast milk from mid-infrared transmission spectroscopy and Pearson’s coefficient correlations between the nutrients and the baseline kit value for TAC, HNE and MDA (*n* = 12).

Nutrient	Mean ± SD	Median	Range	Pearson’s Coefficient Correlations r		
				MDA	4-HNE	TAC
Lipid (g)	4.12 ± 1.4	4.18	1.5–7.3	0.69	0.65	0.46
Protein (g)	1.65 ± 0.3	1.60	1.1–2.2	0.50	0.54	0.33
Lactose (g)	7.93 ± 0.2	7.85	7.6–8.3	−0.31	−0.20	−0.51
Energy (kcal)	78.7 ± 14	78.0	53–111	0.67	0.64	0.43

SD = standard deviation, TAC = total antioxidant capacity, 4-HNE = 4-hydroxynonenal, MDA = malondialdehyde.

## Data Availability

The data presented in this study are available on request from the corresponding author. The data are not publicly available due to the statutory requirements, internal data policies and regulations existing in the collaborating bodies along with the over-arching General Data Protection Regulation (GDPR), the data must be stored in institutional repository (storage platforms).

## References

[1] Breastfeeding WPRO. https://www.who.int/westernpacific/health-topics/breastfeeding.

[2] Nuzzi G., Trambusti I., DI Cicco M.E., Peroni D.G. (2021). Breast Milk: More than Just Nutrition!. Minerva Pediatr..

[3] Friel J.K., Martin S.M., Langdon M., Herzberg G.R., Buettner G.R. (2002). Milk from Mothers of Both Premature and Full-Term Infants Provides Better Antioxidant Protection than Does Infant Formula. Pediatr. Res..

[4] Hård A., Nilsson A.K., Lund A., Hansen-Pupp I., Smith L.E.H., Hellström A. (2019). Review Shows That Donor Milk Does Not Promote the Growth and Development of Preterm Infants as Well as Maternal Milk. Acta Paediatr..

[5] Hanson C., Lyden E., Furtado J., Van Ormer M., Anderson-Berry A. (2016). A Comparison of Nutritional Antioxidant Content in Breast Milk, Donor Milk, and Infant Formulas. Nutrients.

[6] Ballard O., Morrow A.L. (2013). Human Milk Composition: Nutrients and Bioactive Factors. Pediatr. Clin. N. Am..

[7] Andreas N.J., Kampmann B., Mehring Le-Doare K. (2015). Human Breast Milk: A Review on Its Composition and Bioactivity. Early Hum. Dev..

[8] Marnett L.J. (1999). Lipid Peroxidation—DNA Damage by Malondialdehyde. Mutat. Res. Mol. Mech. Mutagen..

[9] Ayala A., Muñoz M.F., Argüelles S. (2014). Lipid Peroxidation: Production, Metabolism, and Signaling Mechanisms of Malondialdehyde and 4-Hydroxy-2-Nonenal. Oxid. Med. Cell. Longev..

[10] Esterbauer H., Eckl P., Ortner A. (1990). Possible Mutagens Derived from Lipids and Lipid Precursors. Mutat. Res. Genet. Toxicol..

[11] Gila-Diaz A., Arribas S.M., Algara A., Martín-Cabrejas M.A., López de Pablo Á.L., Sáenz de Pipaón M., Ramiro-Cortijo D. (2019). A Review of Bioactive Factors in Human Breastmilk: A Focus on Prematurity. Nutrients.

[12] Autor A.P., Frank L., Roberts R.J. (1976). Developmental Characteristics of Pulmonary Superoxide Dismutase: Relationship to Idiopathic Respiratory Distress Syndrome. Pediatr. Res..

[13] Poggi C., Dani C. (2014). Antioxidant Strategies and Respiratory Disease of the Preterm Newborn: An Update. Oxid. Med. Cell. Longev..

[14] Gitto E., Pellegrino S., D’Arrigo S., Barberi I., Reiter R.J. (2009). Oxidative Stress in Resuscitation and in Ventilation of Newborns. Eur. Respir. J..

[15] El-Farrash R.A., El-Shimy M.S., Tawfik S., Nada A.S., Salem D.A.D., Gallo M.S.M., Abd-Elmohsen E.W. (2019). Effect of Phototherapy on Oxidant/Antioxidant Status: A Randomized Controlled Trial. Free Radic. Res..

[16] Perez M., Robbins M.E., Revhaug C., Saugstad O.D. (2019). Oxygen Radical Disease in the Newborn, Revisited: Oxidative Stress and Disease in the Newborn Period. Free Radic. Biol. Med..

[17] Elkabany Z.A., El-Farrash R.A., Shinkar D.M., Ismail E.A., Nada A.S., Farag A.S., Elsayed M.A., Salama D.H., Macken E.L., Gaballah S.A. (2020). Oxidative Stress Markers in Neonatal Respiratory Distress Syndrome: Advanced Oxidation Protein Products and 8-Hydroxy-2-Deoxyguanosine in Relation to Disease Severity. Pediatr. Res..

[18] Negi R., Pande D., Karki K., Kumar A., Khanna R.S., Khanna H.D. (2014). A Novel Approach to Study Oxidative Stress in Neonatal Respiratory Distress Syndrome. BBA Clin..

[19] Hoff D.S., Michaelson A.S. (2009). Effects of Light Exposure on Total Parenteral Nutrition and Its Implications in the Neonatal Population. J. Pediatr. Pharmacol. Ther. JPPT.

[20] Chessex P., Laborie S., Lavoie J.-C., Rouleau T. (2001). Photoprotection of Solutions of Parenteral Nutrition Decreases the Infused Load as Well as the Urinary Excretion of Peroxides in Premature Infants. Semin. Perinatol..

[21] Chessex P., Laborie S., Nasef N., Masse B., Lavoie J.-C. (2017). Shielding Parenteral Nutrition From Light Improves Survival Rate in Premature Infants. J. Parenter. Enter. Nutr..

[22] Hartman C., Shamir R., Simchowitz V., Lohner S., Cai W., Decsi T., Braegger C., Bronsky J., Cai W., Campoy C. (2018). ESPGHAN/ESPEN/ESPR/CSPEN Guidelines on Pediatric Parenteral Nutrition: Complications. Clin. Nutr..

[23] Lapillonne A., Mis N.F., Goulet O., van den Akker C.H.P., Wu J., Koletzko B., Braegger C., Bronsky J., Cai W., Campoy C. (2018). ESPGHAN/ESPEN/ESPR/CSPEN Guidelines on Pediatric Parenteral Nutrition: Lipids. Clin. Nutr..

[24] Unal S., Demirel N., Yaprak Sul D., Ulubas Isik D., Erol S., Neselioglu S., Erel O., Bas A.Y. (2019). The Consequence of Phototherapy Exposure on Oxidative Stress Status of Expressed Human Milk. J. Matern. Fetal Neonatal Med..

[25] van Zoeren-Grobben D., Moison R.M., Ester W.M., Berger H.M. (1993). Lipid Peroxidation in Human Milk and Infant Formula: Effect of Storage, Tube Feeding and Exposure to Phototherapy. Acta Paediatr..

[26] Abcam Lipid Peroxidation (MDA) Assay Kit. https://www.abcam.com/ps/products/-118/ab118970/documents/Lipid-Peroxidation-MDA-assay-protocol-book-v11f-ab118970%20(website).pdf.

[27] Nordic Biosite Human 4-HNE(4-Hydroxynonenal) ELISA Kit. https://nordicbiosite.com/product/EKX-W727S9-96/Human-4HNE4Hydroxynonenal-ELISA-Kit.

[28] Abcam Total Antioxidant Capacity Assay Kit. https://www.abcam.com/ps/products/65/ab65329/documents/-total-antioxidant-capacity-assay-kit-protocol-book-v11-ab65329%20(website).pdf.

[29] Casadio Y.S., Williams T.M., Lai C.T., Olsson S.E., Hepworth A.R., Hartmann P.E. (2010). Evaluation of a Mid-Infrared Analyzer for the Determination of the Macronutrient Composition of Human Milk. J. Hum. Lact..

[30] Cardoso M., Virella D., Papoila A.L., Alves M., Macedo I., E Silva D., Pereira-da-Silva L. (2023). Individualized Fortification Based on Measured Macronutrient Content of Human Milk Improves Growth and Body Composition in Infants Born Less than 33 Weeks: A Mixed-Cohort Study. Nutrients.

[31] Nessel I., De Rooy L., Khashu M., Murphy J.L., Dyall S.C. (2020). Long-Chain Polyunsaturated Fatty Acids and Lipid Peroxidation Products in Donor Human Milk in the United Kingdom: Results From the LIMIT 2-Centre Cross-Sectional Study. JPEN J. Parenter. Enter. Nutr..

[32] Picaud J.C., Steghens J.P., Auxenfans C., Barbieux A., Laborie S., Claris O. (2004). Lipid Peroxidation Assessment by Malondialdehyde Measurement in Parenteral Nutrition Solutions for Newborn Infants: A Pilot Study. Acta Paediatr..

[33] Miloudi K., Comte B., Rouleau T., Montoudis A., Levy E., Lavoie J.-C. (2012). The Mode of Administration of Total Parenteral Nutrition and Nature of Lipid Content Influence the Generation of Peroxides and Aldehydes. Clin. Nutr. Edinb. Scotl..

[34] Păduraru L., Dimitriu D.C., Avasiloaiei A.L., Moscalu M., Zonda G.I., Stamatin M. (2018). Total Antioxidant Status in Fresh and Stored Human Milk from Mothers of Term and Preterm Neonates. Pediatr. Neonatol..

[35] Akdag A., Sari F.N., Dizdar E.A., Uras N., Isikoglu S., Erel O., Dilmen U. (2014). Storage at −80 °C Preserves the Antioxidant Capacity of Preterm Human Milk. J. Clin. Lab. Anal..

[36] Silvestre D., Miranda M., Muriach M., Almansa I., Jareño E., Romero F.J. (2010). Frozen Breast Milk at -20 Degrees C and -80 Degrees C: A Longitudinal Study of Glutathione Peroxidase Activity and Malondialdehyde Concentration. J. Hum. Lact..

[37] Sari F.N., Akdag A., Dizdar E.A., Uras N., Erdeve O., Erel O., Dilmen U. (2012). Antioxidant Capacity of Fresh and Stored Breast Milk: Is −80 °C Optimal Temperature for Freeze Storage?. J. Matern. Fetal Neonatal Med..

